# The effect of letrozole versus artificial hormonal endometrial preparation on pregnancy outcome after frozen-thawed embryos transfer cycles: a randomized clinical trial

**DOI:** 10.1186/s12958-020-00675-z

**Published:** 2020-11-20

**Authors:** Azadeh Hosseini-Najarkolaei, Ashraf Moini, Ladan Kashani, Maryam Farid Mojtahedi, Elnaz Hosseini-Najarkolaee, Ensieh Salehi

**Affiliations:** 1 Department of Gynecology and Obstetrics, Arash women’s Hospital, Tehran University of Medical Sciences, Tehran, Iran; 2 Breast Disease Research Center (BDRC), Tehran University of Medical Sciences, Tehran, Iran; 3 Department of Endocrinology and Female Infertility, Reproductive Biomedicine Research Center, Royan Institute for Reproductive Biomedicine, ACECR, Tehran, Iran; 4 Infertility Ward, Arash Women’s Hospital, Tehran University of Medical Sciences, Tehran, Iran; 5 Laparoscopic Research Centre, Department of Obstetrics and Gynecology, Shiraz University of Medical Sciences, Shiraz, Iran; 6 Department of Obstetrics and Gynecology, Shiraz University of Medical Sciences, Shiraz, Iran; 7 Fertility and Infertility Research Center, Hormozgan University of Medical Sciences, Bandar Abbas, Iran

**Keywords:** Frozen-thawed embryo transfer, Letrozole, Endometrial preparation, Artificial cycle, Ongoing pregnancy rate

## Abstract

**Background:**

Considering that clinical trial studies are limited in polycystic ovary syndrome (PCOS) patients, and there is no consensus on an optimum endometrial preparation protocol for frozen embryo transfer (FET), the present study was designed as a randomized clinical trial to compare the reproductive outcomes following stimulated cycles with letrozole plus human menopausal gonadotropin (HMG) for endometrial preparation compared with routine AC-FET.

**Methods:**

This randomized controlled trial was carried out on infertile PCOS patients who underwent IVF/ICSI and FET cycles in Arash Women’s Hospital affiliated to Tehran University of Medical Sciences between September 2018 and January 2020. PCOS diagnosis was based on the Rotterdam criteria. Eligible patients were randomly allocated into two groups: stimulated cycle with letrozole plus (HMG) (intervention group) and routine artificial hormonal endometrial preparation (control group).

**Results:**

One hundred seventy-seven infertile patients were recruited for participation in the study. Of these, 57 women were excluded due to non-eligibility for entering the study, and a total of 120 patients were randomly assigned to two study groups. After follow up, the cycle outcomes of 57 patients in the intervention group and 59 patients in the control group were compared. The data analysis showed that the two groups did not have significant differences in fundamental and demographic characteristics. After the intervention, there were no significant differences in implantation rate, chemical, ectopic, and clinical pregnancy rates between groups. Moreover, the rates of miscarriage and ongoing pregnancy were similar between groups (*P* > 0.05).

**Conclusions:**

We found similar pregnancy outcomes with two endometrial preparation methods. Noting that each treatment centre should select the most beneficial and cost-effective method with the least adverse effects for patients, letrozole preparations for FET could be incorporated into possible options; however, establishing this approach as first-line treatment is premature in light of current evidence, and future randomized clinical trials with larger sample sizes are required for widespread application.

**Trial registration:**

The study was also registered in the *Iranian Registry of Clinical Trials on March 20th, 2020.* (IRCT20090526001952N12 at www.irct.ir, registered retrospectively).

## Introduction

Polycystic ovarian syndrome (PCOS) is the most common cause of infertility in women at reproductive ages, with a prevalence of 8–13% [1]. Because there is an increased risk of ovarian hyperstimulation syndrome (OHSS) in these patients, the preferred strategy for retrieved oocytes is the freeze-all policy. Recent advances in vitrification techniques for cryopreservation of embryos, and increased acceptance and application of a selective single embryo transfer strategy, have led to a significant increase in the utilization of FET cycles [2]. The potential advantages of the freeze-all policy consisted of an increase in pregnancy rates, a reduction in treatment costs, and a decrease in OHSS incidences during treatment [3].

Different endometrial preparation regimes have been proposed to increase endometrial receptivity in frozen embryo transfer (FET) cycles; however, no superiority of any regimen in terms of clinical pregnancy or live birth rates has been found yet [4, 5]. Most previous studies focused on the optimal method of endometrial preparation in women with normal ovulatory functions, while few studies compare the different methods of endometrial preparation in women with ovarian dysfunction and PCOS diagnosis [2]. Artificial-cycle FET (AC-FET) is more commonly applied for this group of patients because of easier planning and patient convenience [2]. However, the main disadvantage of this method is the dangerous adverse effects of employed hormones, such as the risk of maternal thromboembolic events and genital malformations in male fetuses [2, 6].

In the first randomized clinical trial, Wright et al. compared the stimulated cycles using recombinant follicle stimulating hormone (rFSH) with artificial cycles and found the comparable pregnancy, implantation, cancellation rates and endometrial thickness [7]. Since then, several studies have compared the reproductive outcomes of endometrial preparation with stimulated cycles using gonadotropin and clomiphene or letrozole or both, with the artificial cycles. Notably, a number of studies have shown positive results involving ovarian stimulation (OS) for endometrial preparation in frozen embryo transfer cycles in PCOS patients [2, 8–12], and some studies have not found any differences compared with hormonal endometrial preparation [13, 14].

Letrozole is a third-generation aromatase inhibitor drug without antagonistic effects on the estrogen receptors and it induces *mono-follicular* development due to maintenance the normal central feedback [15]. Furthermore, a pilot study focusing on comparing letrozole and clomiphene citrate for ovulation induction in PCOS women concluded that letrozole positively influences several markers of endometrial receptivity compared with clomiphene citrate [16]. Recently, Zhang et al. performed a retrospective study in patients with PCOS undergoing FET and reported that letrozole administration for endometrial preparation was associated with higher live birth rates compared with artificial cycles after statistical adjustment for confounding factors; therefore, future prospective randomized studies are required to verify these findings [2]. The formation of corpus luteum and appropriate luteal phase support along with utilization of letrozole is another advantage of aromatase inhibitor therapy. Letrozole is relatively safe during pregnancy, and no congenital anomalies were reported for pregnant women who received letrozole. Considering that the clinical trial studies are limited in PCOS patients and given the controversial nature of this subject, the present study was designed as a randomized clinical trial to compare the reproductive outcomes following stimulated cycles with letrozole plus human menopausal gonadotropin (HMG) for endometrial preparation with routine AC-FET.

## Methods

### Study design and population

This randomized controlled trial was carried out on infertile PCOS patients who underwent IVF/ICSI and frozen embryo transfer in Arash Women’s Hospital affiliated to Tehran University of Medical Sciences between September 2018 and January 2020. The study was approved by the Ethics Committee, Tehran University of Medical Sciences (approval ID: IR.TUMS.MEDICINE.REC.1398.834). The study was also registered in the *Iranian Registry of Clinical Trials* (www.irct.ir; IRCT20090526001952N12).

PCOS diagnosis was based on the Rotterdam criteria as fulfilling at least two of the three following items: i) oligo-anovulation or anovulation; ii) clinical or biochemical signs of hyperandrogenism, and iii) PCO morphology on ultrasound, as defined by at least one ovary with volume ≥ 10 cm^3^ or ≥ 12 follicles. Patients over 35 years old, uterine factors, severe male factor infertility, severe endometriosis, immunologic disorders, candidates for preimplantation genetic detection, history of recurrent miscarriage or repeated implantation failure, basal FSH > 10 IU/ml and body mass index (BMI) ≥ 30 kg/m^2^, were excluded from the study.

### Endometrial preparation

The eligible patients were randomly allocated into two groups: cycle stimulated with letrozole plus human menopausal gonadotropin (HMG) (intervention group) and routine artificial hormonal endometrial preparation (control group). Permuted block randomization was conducted by the methodological advisor according to a computer-generated list. The patients’ enrolment and assignment to intervention and control groups were carried out by a researcher midwife in the clinic. Each patient participated in the study only once and on the condition of written consent.

In the intervention group, letrozole (2.5 mg: letrofome*, Iran* Hormone Manufacturing Co., Tehran, *Iran*) was administered twice daily for 5 consecutive days from the third day of the menstrual cycle and then continued with the HMG dose of 75–150 units (Karma Pharmatech GmbH) daily from the fifth day to the ninth. On the tenth day of the spontaneous or discontinued progesterone menstrual cycle, the monitoring transvaginal ultrasound was performed. Endometrial thickness and size of the dominant follicle were measured if the endometrial thickness was less than 7 mm, and the follicle with 12 to 13 mm diameter was observed, the serum LH was measured. The stimulation was continued, and transvaginal ultrasound monitoring was performed every 3 days. When the dominant follicle with 14–15 mm diameter was observed, the serum LH was measured again and, if it was above 10 or more than 180% of the baseline rate, the cycle was cancelled. Whenever the endometrium was ≥8 mm or dominant follicle > 20 mm, human chorionic gonadotropin ampules (HCG) 10,000 IU (*Choriomon,* 5000 *IU,* i.m.*, IBSA company*) were injected for the final oocyte triggering. On day 20, if the endometrial thickness was below 7 mm, the cycle was cancelled regardless of the dominant follicle size.

In the artificial cycle, on days 2–3 of the spontaneous or discontinued progesterone menstrual cycle, an ultrasound scan and E_2_ measurement was carried out to confirm pituitary desensitization and if endometrial thickness was less than 5 mm and serum oestradiol level was less than 50 pg/ml, endometrial preparation was started using 4 mg estradiol valerate daily for 3 days then increased to 6 mg. After 7 days of oestradiol administration, if favourable thickness of endometrium (≥7 mm) was confirmed by ultrasound, estradiol valerate was continued with the same dose, and 50 mg progesterone (*Aburaihan* Pharmaceutical *Co*., *Tehran*, *Iran*) was administered intramuscularly for 2 or 3 days and the day of embryo transfer was determined based on the embryo stage. Otherwise, the oestradiol dosage was increased to 8 mg/day until achievement of the appropriate endometrial thickness. All sonographic evaluations were performed by an expert gynaecologist (A.H) using a Philips Affiniti 70 ultrasound machine with a C10-3v Pure-Wave endo-vaginal probe.

The process of IVF/ICSI and technique of the cryopreservation and thawing of embryos were performed in a standard way similarly for all patients. In short, the embryo quality was evaluated according to Cummins et al.‘s criteria [17] by detecting the number and regularity of blastomeres and the degree of fragmentation on the third day. All top-quality embryos were frozen using the vitrification method on the third day after ovum pick-up. The vitrification procedure was carried out by the Cryotop carrier system (Kitazato Biopharma Co.). The luteal phase was supported by administration of vaginal progesterone twice a day (Suppositories, Cyclogest 400 mg, *Actoverco*). According to the women’s age and embryo quality, up to two top-quality frozen embryos were thawed and transferred at the cleavage stage. In the artificial group, hormone therapy was continued until a pregnancy test was performed, and in case of a positive pregnancy, administration of estradiol valerate and progesterone were continued until 10 weeks of gestation. All pregnant women were followed up on until 21 weeks after ET.

### Outcome measures

The primary outcomes were implantation and chemical and clinical pregnancy rates. The secondary outcomes consist of early miscarriage and ongoing pregnancy rates. Implantation rate was calculated as the number of observed gestational sacs divided by the number of embryos transferred for each patient. Clinical pregnancy was detected by the presence of a gestational sac with fetal heartbeat on the vaginal ultrasound. The spontaneous loss of a clinical pregnancy between 14 and 20 weeks of gestation was considered a late miscarriage. The ongoing pregnancy rate was defined as pregnancies continued for at least 21 weeks after ET and confirmed by the ultrasound scan.

### Calculation of sample size

The sample size was calculated by using the following statistical formula assuming z_α/2_ = 1.96 and z_β_ = 0.85. A sample size of 60 patients was required in each group at a significance level (alpha level) of 0.05 and a power of 80.

$$ n=\frac{\frac{z^2\times p\ \left(1-p\right)}{e^2}}{1+\frac{\Big({z}^2\times p\ \left(1-p\right)}{e^2\ N}} $$

### Statistical analysis

Statistical analysis was conducted using the Statistical Package for Social Sciences (SPSS Inc., Chicago, IL, USA) version 23.0. The continuous variables were compared between groups by *student’s t-test* and presented as mean ± standard deviation (SD). The chi-square test was applied for comparing the categorical variables between groups, and the results were reported as numbers/percentages. The statistically significant level was considered at *p*-value < 0.05.

## Results

**Fig. 1:**
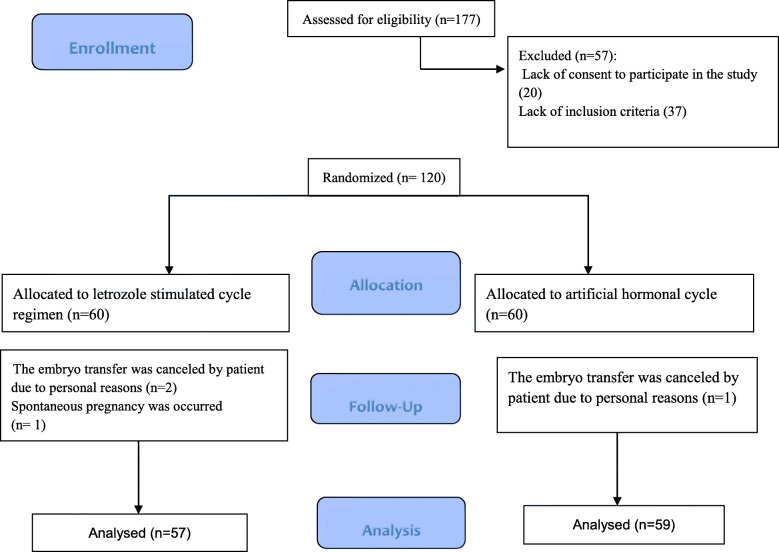
The study flowchart

The flow chart of the study subjects’ sampling according to the Consolidated Standards of Reporting Trials (CONSORT) guideline was as shown in Fig. 1. During the study period, 177 infertile patients were evaluated for participation in the study. Of these, 57 women were excluded due to non-eligibility for entering the study, and a total of 120 women were randomly assigned to either the artificial cycle group (*n* = 60) or stimulated cycle with letrozole + HMG (*n* = 60). After follow up, the cycle outcomes of 57 patients in the intervention group and 59 patients in the control group were compared (Fig. 1). The baseline characteristics of patients are presented in Table 1, and according to the results, the two groups did not show significant differences in terms of demographic and baseline characteristics.

**Table 1:** Comparison of demographic and clinical characteristics of study participants between groups

Variables	Artificial cycle (*n* = 59)	Simulated cycle by letrozole +HMG (*n* = 57)	*P*-value
Age (years)	29.45 ± 0.42	30.12 ± 0.33	0.21
BMI (kg/m^2^)	26.10 ± 0.48	25.70 ± 0.46	0.55
Duration of infertility (Years)	2.80 ± 0.21	3.10 ± 0.26	0.38
Basal serum FSH level (IU/l)	5.86 ± 0.11	5.52 ± 0.13	0.06
Basal serum LH level (IU/l)	7.23 ± 0.37	7.43 ± 0.28	0.65
Serum AMH level (ng/ml)	6.87 ± 0.48	7.52 ± 0.49	0.35
No. of retrieved oocytes of previous COH	21.70 ± 0.96	22.40 ± 0.96	0.61

Descriptive data were presented as Mean ± SD. P-value≤0.05 was considered statistically significant *BMI* Body mass index, *No*. number, *FSH* Follicle-stimulating hormone, *LH* Luteinizing hormone, *AMH* Anti-Müllerian hormone, *COH* Controlled ovarian stimulation, *HMG* Human menopausal gonadotropin

The cycle characteristics and pregnancy outcomes were compared between groups in Table 2. There were no significant differences in implantation rate, chemical, ectopic and clinical pregnancy rates between groups. Moreover, the rates of miscarriage and ongoing pregnancy were similar between groups. The rate of cycle cancellation due to inappropriate endometrium in the stimulated cycle was higher than that of the artificial cycle groups; however, this difference was not significant between groups (*P* = 0.29). Two cases of cycle cancellation in the stimulated cycle group occurred due to unexpected LH rise midcycle or failure to reach an optimum endometrial thickness or both. Despite the recommendation for contraception use, a case of heterotopic pregnancy occurred in the letrozole+HMG group. Laparoscopic surgery was done to remove ectopic pregnancy, and the subject eventually had a successful live birth (Table 2).

**Table 2:** Comparison of cycles and pregnancy outcomes between groups

	Artificial cycle (*n* = 59)	Simulated cycle by letrozole +HMG (*n* = 57)	*P*-value
Endometrial thickness at ET day (mm)	9.92 ± 0.19	9.45 ± 0.16	0.065
Cycle cancellation rate (%)	1 (1.6)	3 (5.2)	0.29
Implantation rate (%)	20%	22%	0.67
Chemical pregnancy rate (%)	25 (42.3)	28 (49.1)	0.46
Clinical pregnancy rate (%)	22 (37.2)	24 (42.1)	0.59
Ectopic pregnancy	1 (1.6)	2 (3.5)	0.53
Heterotopic pregnancy	0 (0)	1 (1.75)	0.30
Miscarriage rate (%)	2 (3.3)	2 (3.5)	0.97
Ongoing pregnancy rate (%)	19 (32.2)	20 (35)	0.74

*HMG* Human menopausal gonadotropin. *P*-value≤0.05 was considered statistically significant

## Discussion

The results of the present study showed similar pregnancy outcomes and cancellation rates after vitrified–warmed ET in both endometrial preparation methods in PCOS patients. Therefore, since minimally stimulated cycles have fewer side effects, it can be suggested as an alternative method. Especially considering recent studies have shown a plausible association between the absence of corpus luteum in FET cycles and adverse obstetrical outcomes [18], the application of endometrial preparation with minimally stimulated cycles is preferable.

To the best of our knowledge, five retrospective [2, 11, 12, 19, 20] and two clinical trial studies [9, 14] have evaluated the effects of letrozole utilization for endometrial preparation before FET; however, only one of the previously published clinical trials [14] possessed a registration number in the specific clinical trial cites. The recent retrospective study, by Zhang et al. in 2019 evaluated a total of 2664 patients with PCOS undergoing FET cycles, reporting that letrozole stimulation during FET cycles significantly improved live birth rates with a decrease in the pregnancy loss rate.

Similarly, three retrospective studies in China and one in Egypt found higher implantation, clinical, ongoing and live birth rates in letrozole stimulated cycles compared with AC [11, 12, 19] and natural cycles [20]. Elsewhere, Peigné et al. (2019) in a retrospective study concluded that live birth rate was significantly higher with mild OS than with the AC preparation, even after adjusting for potential confounders; suggesting that the first-line endometrial preparation could be OS instead of an AC and a potential defect of the luteal phase may exist in AC preparation [21]. However, Aleyasin et al. in a randomized clinical trial found no significant difference in terms of live birth rate between the letrozole plus HMG method and the artificial FET protocol [14]. In another clinical trial by Tahoon et al. it was reported that using letrozole stimulated cycles for endometrial preparation in cryopreserved ET yields a significantly higher ongoing pregnancy rate than artificial cycles [9]. An overview of past studies reveals that the use of letrozole for endometrial preparation can be advantageous. Two possible reasons for the observed qualities of the letrozole approach were proposed *which is as follows*: i) letrozole decreases intraovarian and serum estrogen levels by blocking the conversion of androgens to estrogens in the ovarian granulosa cells [20], subsequently low estrogen levels reduce ubiquitination of estrogen receptors, this process leads to faster endometrial proliferation and increased blood level in the uterus and endometrium, with positive effects on pregnancy outcomes [19] and ii) there is some evidence that letrozole may potentially improve endometrial receptivity [2]. In this way, Miller et al. concluded that the lack of endometrial αvβ*3* integrin expression is associated with a poor prognosis outcome in IVF cycles that might be improved with letrozole co-treatment [22]. Elsewhere, Ganesh et al., in a preliminary study reported that OS with the use of letrozole was associated with a significant increase in the epithelial and stromal expression of uterine receptivity markers, including integrin, leukaemia inhibitory factor, and L-selectin, in women with unexplained infertility compared with natural cycles [23]; all of which may have positive effects on the pregnancy rate after ET [2].

Meanwhile, we observed a slight increase in the endometrial thickness on the day of ET in the letrozole-treated group. Similarly, Zhang et al. found the endometrial thickness was significantly greater in the letrozole group than in the AC group, both on the day of starting progesterone and on the day of ET [2]. However, some studies found no significant difference [14, 21] or even higher endometrial thickness after the artificial preparation method [9]. Therefore, more randomized trials with higher sample size are still required to conclude and comment on this regards.

In terms of the long-term safety of fertility treatment for future offspring, Tatsumi et al. in a recent population-based study suggested that the administration of letrozole during IVF cycles neither increased the risk of major congenital anomalies nor compromised neonatal outcomes of the newborns compared with natural cycles [24].

Noting that this study is the largest randomized clinical trial design at the time of writing; yet, this trial could not find a significant effect to prioritize the use of letrozole for endometrial preparation over the hormonal method, which may be due to sample size limitation. Therefore, we suggest that a multi-centre clinical trial with a larger sample size be conducted in this field. Another limitation is that we did not assay the endometrial receptivity markers on both groups of patients, so further studies on this subject are warranted to explore the mechanism of the effect of letrozole on the endometrium.

In conclusion, we found similar pregnancy outcomes after evaluating two endometrial preparation methods. Noting that each treatment centre should select the most beneficial and cost-effective method with the least adverse effects for patients, letrozole preparations for FET could be incorporated into possible options; however, establishing this approach as first-line treatment is premature in light of current evidence, and future randomized clinical trials with larger sample sizes are required for widespread application.

## Data Availability

The datasets used or analyzed during the current study are available from the corresponding authors on reasonable request.

## Abbreviations

FET: Frozen embryo transfer
PCOS: Polycystic ovary syndrome
OHSS: Ovarian hyperstimulation syndrome
rFSH: Recombinant follicle stimulating hormone
AC: Artificial-cycle
E_2_: Estradiol
HMG: Human menopausal gonadotrophins
hCG: Human chorionic gonadotropin
SD: Standard deviation
